# HMBOX1 interacts with MT2A to regulate autophagy and apoptosis in vascular endothelial cells

**DOI:** 10.1038/srep15121

**Published:** 2015-10-12

**Authors:** HanLin Ma, Le Su, HongWei Yue, XiaoLei Yin, Jing Zhao, ShangLi Zhang, HsiangFu Kung, ZhiGang Xu, JunYing Miao

**Affiliations:** 1 Shandong Provincial Key Laboratory of Animal Cells and Developmental Biology, School of Life Science, Shandong University, Jinan 250100, China; 2 The Key Laboratory of Cardiovascular Remodeling and Function Research, Chinese Ministry of Education and Chinese Ministry of Health, Shandong University Qilu Hospital, Jinan, 250012, China; 3 Institute of Pathology and Southwest Cancer Center, Third Military Medical University, Chongqing, 400038, China

## Abstract

We previously found that Homeobox containing 1 (HMBOX1) was required for bone mesenchymal stem cell (BMSC) and mouse embryonic stem cell (ESC) differentiation into vascular endothelial cells (VECs). However, the function of HMBOX1 in VECs is still unknown. In this study, we found that HMBOX1 was abundantly expressed in the cytoplasm of human umbilical vascular endothelial cells (HUVECs). Knockdown of HMBOX1 induced apoptosis and inhibited autophagy. Overexpression of HMBOX1 inhibited apoptosis induced by fibroblast growth factor 2 deprivation and promoted autophagy. Metallothionein 2A (MT2A) was identified as an interaction protein with HMBOX1 by yeast two-hybrid assay, and confirmed by co-immunoprecipitation. Overexpression of HMBOX1 elevated intracellular free zinc level. Knockdown of MT2A inhibited this phenomenon. Moreover, N,N,N = ,N = -tetrakis (2-pyridylmethyl) ethylenediamine (TPEN), a zinc chelator, reversed the anti-apoptosis and pro-autophagy effects of HMBOX1. In conclusion, HMBOX1 regulated intracellular free zinc level by interacting with MT2A to inhibit apoptosis and promote autophagy in VECs.

Homeobox containing 1 (HMBOX1), first identified and isolated from the human pancreatic cDNA library, was first known as a transcription repressor^1^. Although it is abundantly expressed in pancreas and moderately in brain, placenta, prostate, thymus, and testes, we have few reports about the functions of HMBOX1. In natural killer cells, HMBOX1 was a negative regulator of cell functions via NKG2D/DAP10. Subsequently, this group found that HMBOX1 displayed its effect as a transcription repressor on the transcription activity of interferon γ (IFN-γ) promoter in natural killer cells^2^. Recently, HMBOX1 was found to bind double-stranded telomere repeats and associate with the active telomerase complex to support telomerase-dependent telomere elongation^3^. Moreover, HMBOX1 participated in telomere maintenance in alternative lengthening of telomere (ALT) cells, which extended their telomeres independent of telomerase^4^.

Metallothioneins (MTs) are a family of small proteins containing a high proportion of cysteines and have high affinity with heavy metals such as zinc and calcium. Four isoforms have been identified (MT-I–IV)^5^. Metallothionein 2A (MT2A) is the most prominent in endothelial cells. MT2A is involved in endothelial cell proliferation and migration^6^. However, we lack data on the relationship between HMBOX1 and MT2A in HUVECs.

In our previous study, we found that the level of HMBOX1 was upregulated in the differentiation of bone marrow mesenchymal stem cells (BMSCs) and mouse embryonic stem cells (ESCs) into endothelial cells (ECs) induced by a small molecular 6-amino-2,3-dihydro-3-hydroxymethyl-1,4-benzoxazine (ABO). Moreover, ABO failed to induce the formation of ECs without HMBOX1^7^,^8^. Since HMBOX1 is necessary for VEC differentiation, we speculate that HMBOX1 is highly expressed in normal VECs and plays an important function in VECs. Here, we investigated the distribution and function of HMBOX1 in HUVECs and found that HMBOX1 elevated the level of intracellular free zinc by interacting with MT2A to maintain HUVEC survival.

## Results

### HMBOX1 was abundantly expressed in cytoplasm of HUVECs and inhibited HUVEC apoptosis

**Figure 1:**
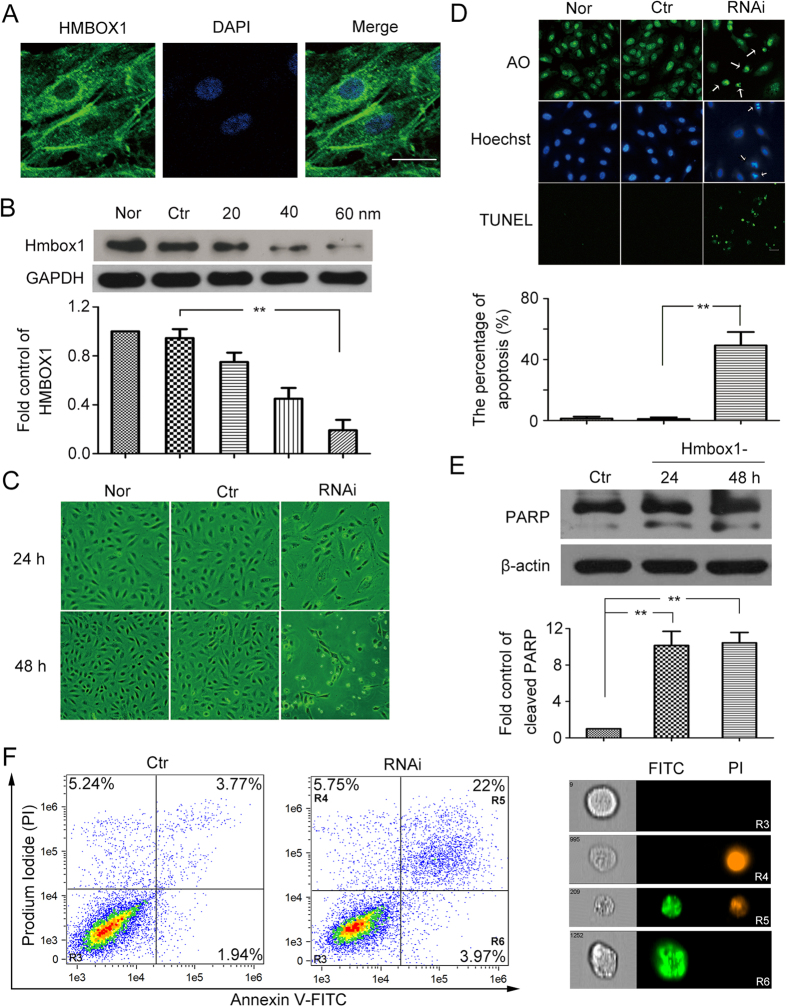
Knockdown of Homeobox containing 1 (HMBOX1) induced human umbilical vascular endothelial cell (HUVEC) apoptosis. (**A**) Subcellular distribution of HMBOX1 in normal HUVECs. Scale bar: 16 μm. (**B**) Western blot analysis of siRNA knockdown of HMBOX1 (20, 40 or 60 nM) or scramble siRNA (60 nM; Ctr) for 24 h in HUVECs. HMBOX1 levels are relative to that of GAPDH. Nor, HUVECs cultured in normal M199 medium. (cropped, full-length blots are in Supplementary Fig. S1) (**C**) Morphological changes of HUVECs treated with 60 nM siRNA of HMBOX1 for 24 or 48 h (20×). RNAi, HUVECs transfected with HMBOX1 siRNA (60 nM). (**D**) Acridine orange (AO), Hoechst 33258 and TUNEL staining of apoptotic HUVECs. Arrows indicate apoptotic cells and typical nuclear fragmentation. Scale bar: 16 μm. The proportion of apoptosis was quantified by TUNEL staining at 24 h. (**E**) Western blot analysis of cleaved forms of PARP in HUVECs transfected with HMBOX1 siRNA for 24 or 48 h. The level of cleaved PARP was relative to that of β-actin. (cropped, full-length blots are in Supplementary Fig. S1). (Data are mean ± SEM, *p < 0.05, **p < 0.01, n = 3). (**F**) AnnexinV-FITC/PI staining and flow cytometry assay to determine the proportion of apoptosis after HMBOX1 knockdown for 24 h. Images showed the typical morphologies of cells in the different regions.

We first detected the subcellular distribution of endogenous HMBOX1 in normal HUVECs. Immunocytochemistry assay showed HMBOX1 abundantly expressed in cytoplasm, with filamentary distribution in HUVECs (Fig. 1A). Small interfering RNA (siRNA) was used to knock down the level of HMBOX1 in HUVECs. SiRNA knockdown could effectively decrease the protein level of HMBOX1. The concentration of 60 nM had the best efficiency (Fig. 1B), so we used 60 nM siRNA in the following knockdown experiments. After HMBOX1 siRNA knockdown for 24 h, some cells detached from the substratum and became round. After treatment for 48 h, the number of cells adhered to the substratum significantly decreased; most cells became round and appeared apoptotic (Fig. 1C). With HMBOX1 siRNA knockdown, cells showed nuclear condensation and fragmentation, characteristics of apoptosis by the acridine orange (AO) and Hoechst stainings (Fig. 1D). About 49.3% cells were TUNEL-positive, which was significantly higher than in the control group (1.04%) (Fig. 1D). Moreover, western blot assay of the protein level of cleaved Poly (ADP-ribose) polymerase (PARP), a hallmark of caspase-3 activation and apoptosis, corroborated the induced apoptosis with HMBOX1 siRNA knockdown (Fig. 1E). In addition, HMBOX1 knockdown induced cleaved caspase-8 level, but not cleaved caspase-9 level as compared with scramble siRNA treatment (Supplementary Fig. S2). We also used AnnexinV-FITC/PI staining and flow cytometry assay to determine the the extent of apoptosis after HMBOX1 knockdown. It is revealed that the proportion of apoptotic cells was higher in HMBOX1 RNAi group than control group (25.97% vs. 5.71%) (Fig. 1F). Thus, knockdown of HMBOX1 might trigger extrinsic apoptotic pathway in HUVECs.

**Figure 2:**
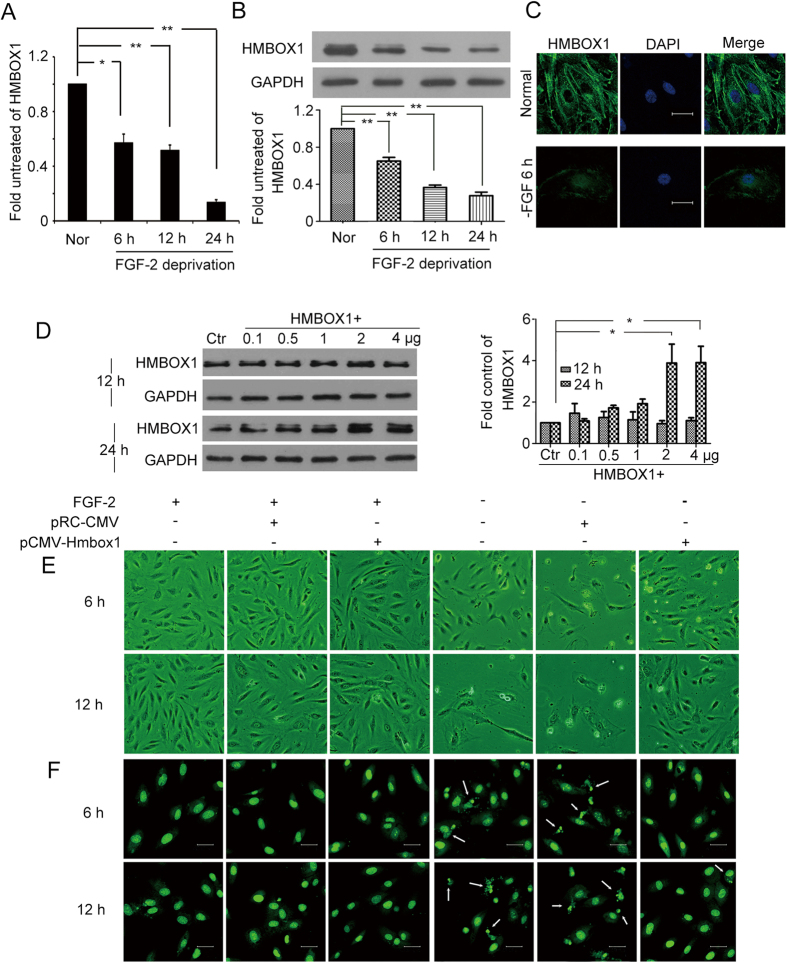
The level of HMBOX1 decreased in HUVECs deprived of fibroblast growth factor 2 (FGF-2). (**A**) Quantitative PCR (qPCR) analysis of the mRNA level of *HMBOX1* and (**B**) western blot analysis of the protein level of HMBOX1 in HUVECs cultured in basal M199 medium with FGF-2 deprivation for 6, 12 and 24 h. Protein levels are relative to that of GAPDH. (cropped, full-length blots are in Supplementary Fig. S3). (Data are mean ± SEM, *p < 0.05, ** *p* < 0.01, n = 3). (**C**) Immunofluorescence images of HMBOX1 in normal cells and cells deprived of FGF-2 for 6 h under confocal microscopy. Images are representative of three independent experiments. DAPI staining of nuclei was in blue. Scale bar: 16 μm. (**D**) Western blot analysis of the transfection efficiency of HMBOX1. Ctr, HUVECs transfected with pRC-CMV for 24 h; HMBOX1+, HUVECs transfected with pCMV-HMBOX1 (0.1, 0.5, 1, 2 or 4 μg) for 12 or 24 h. Relative level of HMBOX1 to that of GAPDH. (*p < 0.05, **p < 0.01, n = 3). HUVECs were transfected with pRC-CMV or pCMV-HMBOX1 for 24 h, then cultured in the basal M199 medium without FGF-2 for 6 or 12 h. (cropped, full-length blots are in Supplementary Fig. S3) (**E**) Microscopy images of the morphological changes of HUVECs. (20×). (**F**) Acridine orange (AO) staining of the nuclear condensation and fragmentation of HUVECs. Arrows indicate apoptotic cells and typical nuclear fragmentation. Scale bars: 20 μm. Images are representative of at least 3 independent experiments.

**Figure 3:**
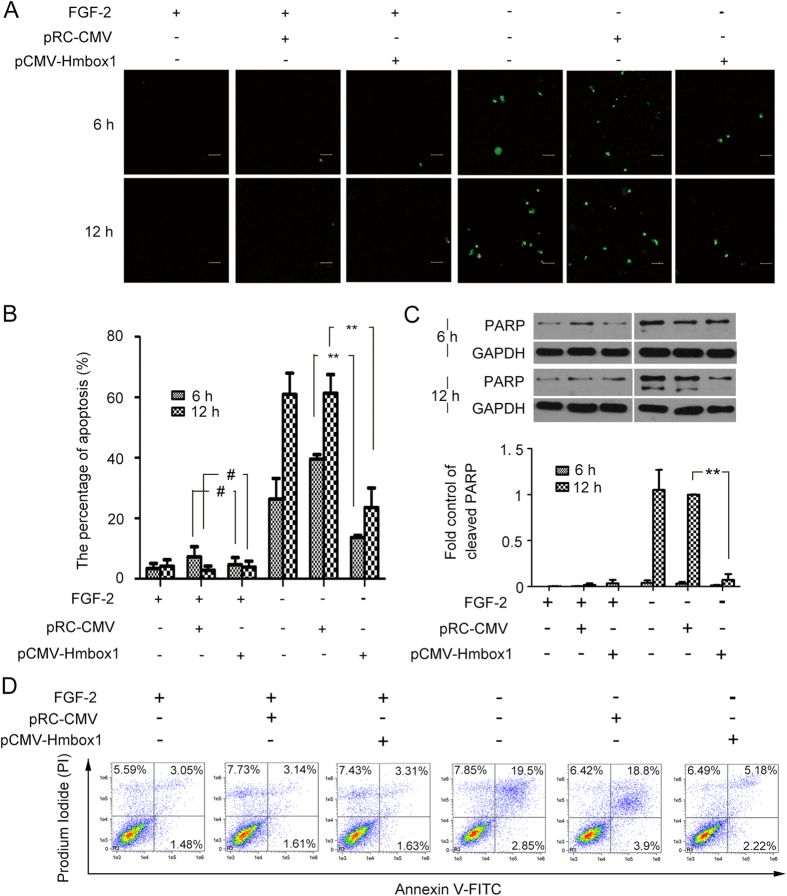
HMBOX1 inhibited HUVEC apoptosis induced by FGF-2 deprivation. (**A**) Nuclear fragmentation of cells by TUNEL staining. Cells were transfected with pRC-CMV or pCMV-HMBOX1 for 24 h, then cultured in basal medium deprived of FGF-2 for 6 or 12 h. Scale bar: 16 μm. (**B**) Proportion of apoptosis quantified by TUNEL staining. (#*p* > 0.05, **P < 0.01, n = 3) (**C**) Western blot analysis of cleaved PARP level in HUVECs. The level of cleaved PARP is relative to that of GAPDH. Cells were transfected with pRC-CMV or pCMV-HMBOX1 for 24 h, then cultured in basal medium deprived of FGF-2 for 6 or 12 h. (cropped, full-length blots are in Supplementary Fig. S4). (Data are mean ± SEM, **p < 0.01, n = 3). (**D**) AnnexinV-FITC/PI staining and flow cytometry assay to determine the proportion of apoptosis after HMBOX1 overexpression with FGF-2 or without FGF-2 for 12 h.

It is well known that HUVECs will take apoptosis after deprived of fibroblast growth factor 2 (FGF-2). And knockdown of HMBOX1 would induce HUVEC apoptosis. Thus, deprivation of FGF-2 was used as an apoptosis model to examine whether the expression of HMBOX1 was decreased during this model and whether the overexpression of HMBOX1 could inhibit HUVEC apoptosis induced by FGF-2 deprivation. After HUVECs became apoptotic on FGF-2 deprivation, both the mRNA and protein levels of *HMBOX1* were time-dependently decreased in HUVECs (Fig. 2A–C). Then, we overexpressed HMBOX1 in HUVECs by transfecting the plasmid pCMV-HMBOX1 containing the full-length HMBOX1 cDNA. Western blot analysis confirmed the transfection efficiency (Fig. 2D). The concentration of 2 μg/ml for 24 h had the best transfection efficiency. Thus, in the following experiments, we used 2 μg/ml pCMV-HMBOX1 to overexpress HMBOX1 in HUVECs. HUVECs were transfected with pCMV-HMBOX1 for 24 h, and then deprived of FGF-2 for 6 or 12 h to induce apoptosis. Cells transfected with pRC-CMV were apoptotic; most cells detached from the substratum and became round. However, apoptosis was inhibited in cells transfected with pCMV-HMBOX1 (Fig. 2E). Acridine orange (AO) staining revealed that HMBOX1 overexpression significantly inhibited HUVEC apoptosis (Fig. 2F): cells showed decreased nuclear condensation and fragmentation (arrows) after transfection with pCMV-HMBOX1 as compared with pRC-CMV. Moreover, the number of TUNEL-positive cells was reduced with HMBOX1 overexpression (Fig. 3A). The proportion of apoptotic cells decreased to 6.8% and 11.8% with pCMV-HMBOX1 after FGF-2 deprivation for 6 and 12 h respectively, from 19.8% and 30.7%, respectively, with pRC-CMV (Fig. 3B). Western blot assay of cleaved PARP corroborated the anti-apoptosis effect of HMBOX1 in HUVECs (Fig. 3C). In addition, the results from AnnexinV-FITC/PI staining and flow cytometry assay also showed that the proportion of apoptotic cells decreased to 7.4% with pCMV-HMBOX1 after FGF-2 deprivation for 12 h, from 22.7% with pRC-CMV (Fig. 3D). Thus, HMBOX1 could maintain HUVEC survival.

### HMBOX1 promotion of autophagy in HUVECs depended on mammalian target of rapamycin (mTOR)

**Figure 4:**
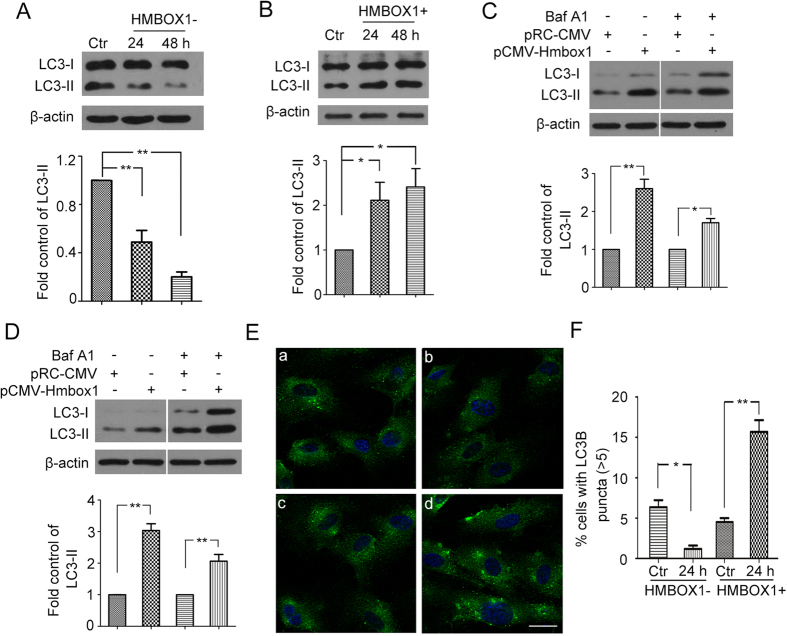
HMBOX1 promotion of autophagy in HUVECs. (**A**) Western blot analysis of protein levels of LC3-I and LC3-II in HUVECs treated with scramble siRNA (Ctr) or HMBOX1 siRNA (HMBOX1-) for 24 or 48 h. Levels of LC3-I and LC3-II are relative to that of β-actin, (cropped, full-length blots are in Supplementary Fig. S5). (**B**) Western blot analysis of protein levels of LC3-I and LC3-II in HUVECs transfected with pRC-CMV (Ctr) or pCMV-HMBOX1 (HMBOX1+) for 24 or 48 h. Levels of LC3-II are relative to that of β-actin. (cropped, full-length blots are in Supplementary Fig. S5). Western blot analysis of protein levels of LC3-I and LC3-II in HUVECs transfected with pRC-CMV or pCMV-HMBOX1 for 24 h (**C**) or 48 h (**D**) with or without 50 nM Bafilomycin A1 (Baf A1). Levels of LC3-II are relative to that of β-actin, (cropped, full-length blots are in Supplementary Fig. S5). (**E**) Immunostaining of LC3B in HUVECs. a, HUVECs transfected with scramble siRNA; b, HMBOX1 siRNA; c, pRC-CMV; d, pCMV-HMBOX1. Scale bar: 16 μm. (**F**) Proportion of cells containing LC3B puncta (>5) in (**E**). (Data are mean ± SEM, *p < 0.05, **p < 0.01, n = 3).

**Figure 5:**
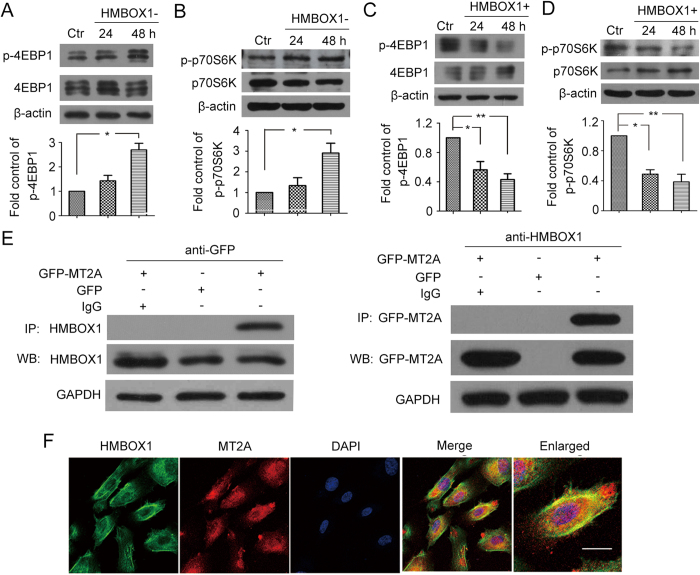
The autophagy induced by HMBOX1 depended on mammalian target of rapamycin (mTOR) and HMBOX1 interacted with metallothionein 2A (MT2A). Western blot analysis of (**A**) protein levels of 4EBP1 and phosphorylated 4EBP1 (p-4EBP1, Thr37/46) and (**B**) protein levels of p70S6K and phosphorylated p70S6K (p-p70S6K, Thr389) in HUVECs transfected with scramble siRNA (Ctr) or HMBOX1 siRNA (HMBOX1-) for 24 or 48 h. Western blot analysis of (**C**) protein levels of 4EBP1 and phosphorylated 4EBP1 (p-4EBP1, Thr37/46) (**D**) protein levels of p70S6K and phosphorylated p70S6K (p-p70S6K, Thr389) in HUVECs transfected with pRC-CMV (Ctr) or pCMV-HMBOX1 (HMBOX1+) for 24 or 48 h. Level of p-4EBP1 is relative to that of 4EBP1,level of p-p70S6K is relative to that of p70S6K. (cropped, full-length blots are in Supplementary Fig. S6). (Data are mean ± SEM, *p < 0.05, **p < 0.01, n = 3) (**E**) Co-immunoprecipitation (Co-IP) of HMBOX1 with GFP-tagged MT2A proteins from HEK293 cells. HEK293 cells were transfected with pEGFP or pEGFP-MT2A for 48 h. (cropped, full-length blots are in Supplementary Fig. S7). (**F**) Double immunocytochemical staining of HMBOX1 (green) and MT2A (red) showing agglomeration and co-localization in HUVECs. Overlay of the HMBOX1 and MT2A with yellow indicates co-localization. Scale bar: 16 μm. Images are representative of at least 3 independent experiments.

In light of the relationship between apoptosis and autophagy, we then examined the effect of HMBOX1 in HUVEC autophagy. Knocking down HMBOX1 for 24 and 48 h reduced the level of autophagic protein microtubule-associated protein 1 light chain 3- II (LC3-II) as compared with scramble siRNA (Fig. 4A). LC3-II accumulation was elevated with pCMV-HMBOX1 overexpression than pRC-CMV treatment (Fig. 4B). To validate the effect of HMBOX1 on autophagy flux, Bafilomycin A1 were used to block the flow of autophagy. HMBOX1 could elevate LC3-II accumulation in the presence of Bafilomycin A1 for 24 h and 48 h, respectively (Fig. 4C,D). These data suggest that HMBOX1 promoted the flow of autophagy in HUVECs. Consistently, immunofluorescence assay showed decreased proportion of cells containing LC3B puncta (>5) in cells transfected with HMBOX1 siRNA and increased in cells with pCMV-HMBOX1 transfection (Fig. 4D,E). Moreover, the phosphorylation of 4E-binding protein 1 (4EBP1) and p70S6K, downstream targets of mTOR, was increased with HMBOX1 siRNA (Fig. 5A,B) and inhibited with pCMV-HMBOX1 transfection (Fig. 5C,D). HMBOX1 promotion of HUVEC autophagy might depend on mTOR.

### HMBOX1 interacted with MT2A

After we found the function of HMBOX1 to maintain cell survival in HUVECs, we were interested in the mechanism by which HMBOX1 inhibits apoptosis and promotes autophagy in HUVECs. Because there were no reports about the function of HMBOX1 in HUVECs and the proteins that interact with HMBOX1, we searched for proteins that might interact with HMBOX1 by performing yeast two-hybrid assay. We generated a fusion construct of the GAL4 DNA-binding domain with the *HMBOX1* used as bait for screening a human liver yeast two-hybrid cDNA library. Out of the 4.8 × 10^5^ transformants screened, 30 clones grew in the absence of tryptophan, leucine, histidine, and adenine and expressed α-galactosidase activity. pACT2 prey plasmids were successfully isolated from the 30 clonies and re-introduced together with the bait plasmid into yeast and tested for the specificity of α-galactosidase expression. After retransformation, 21 independent positive clones were identified and sequenced; one was identified as MT2A (Supplementary Table S1). We verified the interaction between HMBOX1 and MT2A in HEK293 cells (Fig. 5E) and the agglomeration and co-localization of HMBOX1 and MT2A in HUVECs (Fig. 5F).

### HMBOX1 regulated the intracellular free zinc level by interacting with MT2A

**Figure 6:**
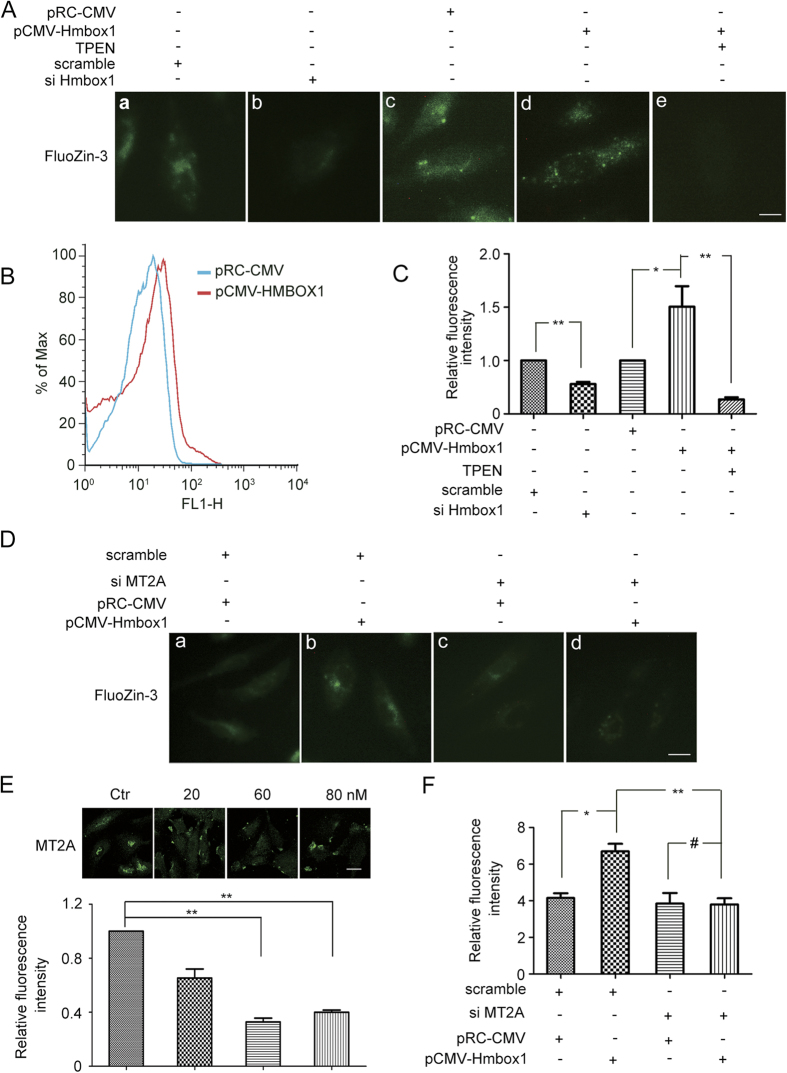
HMBOX1 regulated the intracellular free zinc level by interacting with MT2A. (**A**) Immunofluorescence assay of level of intracellular free zinc regulated by HMBOX1. Images represent fluorescence intensity of FluoZin-3–Zn^2+^ by fluorescence microscopy. HUVECs were transfected with a, scramble control siRNA; b, specific HMBOX1 siRNA; c, pRC-CMV; d, pCMV-HMBOX1; or e, pCMV-HMBOX1, then with N,N,N = ,N = -tetrakis (2-pyridylmethyl) ethylenediamine (TPEN). Scale bar: 16 μm. (**B**) Flow cytometry of the intracellular level of zinc. pRC-CMV, HUVECs transfected with pRC-CMV; pCMV-HMBOX1, HUVECs transfected with pCMV-HMBOX1. Images are representative of at least 3 independent experiments. (**C**) Quantification of relative fluorescence intensity per cell in A. (*p < 0.05, **p < 0.01, n = 3). (**D**) Fluorescence microscopy of FluoZin-3–Zn^2+^. HUVECs were transfected with scramble control siRNA for 24 h, then a, pRC-CMV or b, pCMV-HMBOX1. HUVECs were transfected with specific MT2A siRNA for 24 h, then c, pRC-CMV or d, pCMV-HMBOX1. (**E**) Immunofluorescence images of MT2A in HUVECs. Ctr, HUVECs were transfected with scramble siRNA; 20, 60, 80 nM: HUVECs were transfected with specific MT2A siRNA at 20 nM, 60 nM or 80 nM. Quantification of relative fluorescence intensity per cell. (**P < 0.01, n = 3). Scale bar: 16 μm. (**F**) Quantification of relative fluorescence intensity per cell in D. (#*p* > 0.05, *p < 0.05, **p < 0.01, n = 3).

Because MT2A has a high affinity with zinc and interacted with HMBOX1, we examined whether HMBOX1 affected the level of intracellular free zinc level in HUVECs. After siRNA knockdown and pCMV-HMBOX1 overexpression of HMBOX1, we monitored the intracellular free zinc level by using the specific fluorescent indicator for labile zinc FluoZin-3. HMBOX1 siRNA treatment decreased the level of intracellular free zinc and pCMV-HMBOX1 overexpression increased labile zinc concentration by almost two-fold as compared with pRC-CMV treatment (Fig. 6Aa–d,C). Overexpression of HMBOX1 increased the zinc level was also confirmed by flow cytometry (Fig. 6B). TPEN, a specific zinc chelator inhibited the zinc level induced by HMBOX1 overexpression, which was used as a positive control (Fig. 6Ae,C).

Next we investigated whether MT2A was involved in the zinc regulation. The function of MT2A in HUVECs was blocked by specific siRNA. Immunocytochemistry assay revealed the transfection efficiency (Fig. 6E). Cells with MT2A siRNA knockdown were also transfected with pCMV-HMBOX1 for HMBOX1 overexpression. MT2A siRNA knockdown and pCMV-HMBOX1 overexpression inhibited the elevated level of intracellular free zinc as compared with pCMV-HMBOX1 alone (Fig. 6Db,d,F). And without MT2A, pCMV-HMBOX1 overexpression could no longer increase the zinc level (Fig. 6Dc,d,F). These data suggested that HMBOX1 regulated the level of intracellular free zinc by interacting with MT2A. With MT2A knockdown, the level of intracellular zinc could not be increased by HMBOX1 overexpression in HUVECs.

### By increasing the level of intracellular free zinc, HMBOX1 regulated HUVEC apoptosis and autophagy

**Figure 7:**
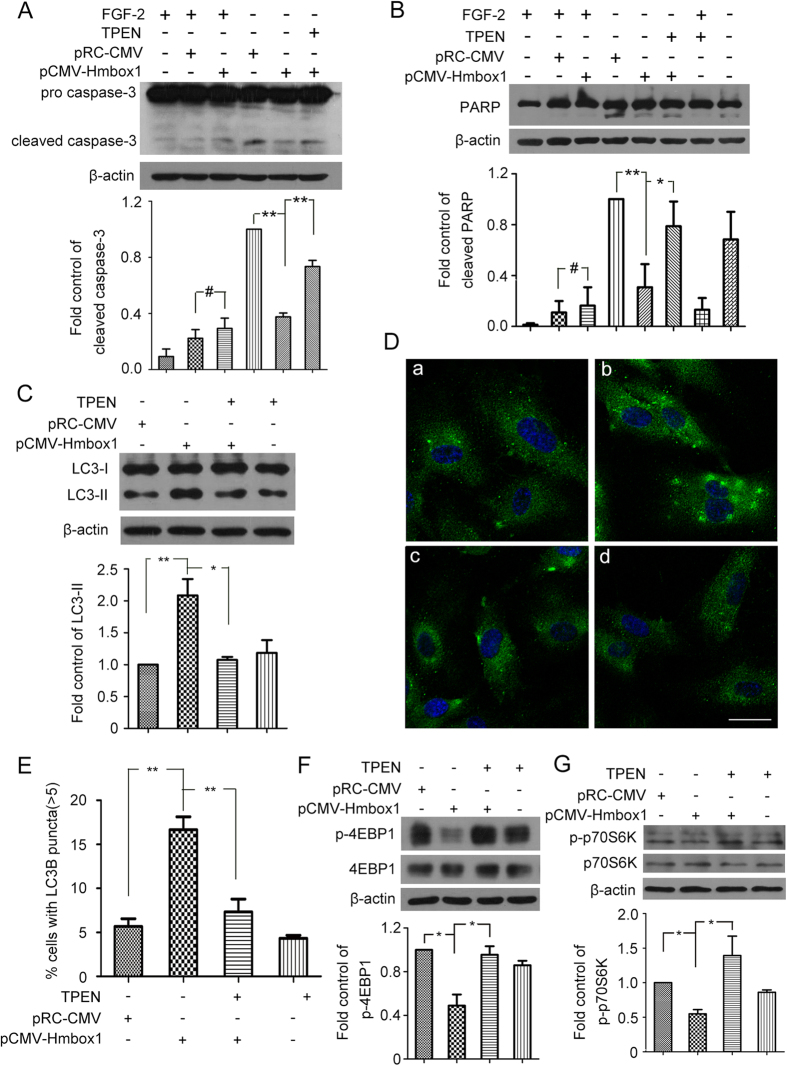
HMBOX1 regulated HUVEC apoptosis and autophagy by increasing the level of intracellular free zinc. Western blot analysis of (**A**) caspase-3 and (**B**) cleaved PARP protein levels with β-actin as a loading control, and quantification. HUVECs were transfected with pRC-CMV or pCMV-HMBOX1 for 24 h, then cultured in basal M199 medium without FGF-2 for 12 h with or without 2.5 μM TPEN. (cropped, full-length blots are in Supplementary Fig. S8) (Data are mean ± SEM, #*p* > 0.05, *p < 0.05, **p < 0.01, n = 3) (**C**) Western blot analysis of protein levels of LC3-I and LC3-II and quantification. HUVECs were transfected with pRC-CMV or pCMV-HMBOX1 for 12 h, then with 2.5 μM TPEN for 12 h. (**D**) Immunostaining of LC3B in HUVECs. HUVECs were transfected with a, pRC-CMV or b, pCMV-HMBOX1 for 24 h; c, HUVECs were transfected with pCMV-HMBOX1 for 12 h, then with 2.5 μM TPEN for 12 h; d, HUVECs were treated with 2.5 μM TPEN for 12 h. Scale bar: 16 μm. (**E**)The proportion of cells containing LC3B puncta (>5) in D. (**P < 0.01, n = 3). Western blot analysis of the protein levels of 4EBP1 or p-4EBP1 (Thr37/46) (**F**) and p70S6K or p-p70S6K (Thr389) (**G**), HUVECs were transfected with pRC-CMV or pCMV-HMBOX1 for 12 h, then treated with 2.5 μM TPEN for 12 h. (cropped, full-length blots are in Supplementary Fig. S9). (Data are mean ± SEM, #*p* > 0.05, *p < 0.05, **p < 0.01, n = 3).

Next we investigated whether decreasing the level of free zinc by TPEN could reverse the HMBOX1–inhibited apoptosis. HUVECs were transfected with pCMV-HMBOX1 for 24 h, then FGF-2–deprived for 12 h to induce cell apoptosis. Overexpression of HMBOX1 reduced cleaved caspase-3 level as compared with pRC-CMV treatment. However, TPEN abrogated this effect (Fig. 7A). The same effect was observed with cleaved PARP level (Fig. 7B). These results suggested that HMBOX1 regulated HUVEC apoptosis by controlling the intracellular level of free zinc.

We examined cell autophagy after TPEN treatment. LC3-II accumulation was greater with pCMV-HMBOX1 than pRC-CMV treatment (Fig. 7C), as was found earlier (Fig. 4B), but reduced with TPEN treatment (Fig. 7C). Consistently, immunofluorescence assay revealed an increased proportion of cells with LC3B puncta (>5) induced by pCMV-HMBOX1, which was reversed with TPEN (Fig. 7D,E). Moreover, pCMV-HMBOX1-decreased phosphorylation of 4EBP1 and p70S6K was increased with TPEN treatment (Fig. 7F,G). HMBOX1 regulated HUVEC autophagy by controlling the intracellular level of free zinc.

## Discussion

In the present study, we demonstrated that endogenous HMBOX1 was abundantly expressed in the cytoplasm of HUVECs. Through interacting with MT2A and regulating intracellular zinc levels, HMBOX1 regulated HUVEC apoptosis and autophagy. To our knowledge, this is the first report about the role of HMBOX1 in the regulation of apoptosis and autophagy.

The endothelium, composed of VECs, is the thin layer of cells that lines the interior surface of blood vessels. It forms an interface between circulating blood in the lumen and vessel wall. VECs, which directly contact with blood^9^, are involved in many aspects of vascular biology, including barrier function, blood clotting, inflammation, angiogenesis and vasoconstriction^10^. Because of these important functions, once endothelial dysfunction occurs, many vascular diseases are induced. Apoptosis, defined as programmed cell death, plays an important role in maintaining cellular homeostasis by regulating cell death with stress^11^. VEC apoptosis could represent a main form of VEC injury, which leads to inflammation in the vessel wall and has been associated with many diseases such as atherosclerosis, hypertension, diabetes mellitus and thrombosis^12^. Autophagy is also known for cellular maintenance by specific degradation and the recycling of damaged organelles and misfolded proteins. Thus, apoptosis and autophagy are 2 evolutionarily conserved processes that maintain VEC homeostasis. Autophagy is mainly considered to have a physiologic cytoprotective effect or to maintain cell survival under stress conditions^13^. Current evidence suggests that autophagy may mediate resistance to apoptosis. Moderate autophagy is necessary for cell survival, whereas cell apoptosis would appear with completely uncontrolled or blocked autophagy^14^,^15^.

In this study, HMBOX1 siRNA knockdown inhibited autophagy in HUVECs, which resulted in cell apoptosis. Thus, autophagy inhibition accelerated apoptosis, although this theory is controversial^16^. In normal HUVECs, HMBOX1 may maintain a certain level of autophagy to maintain cell survival. HMBOX1 might be an important factor in investigating the crosstalk between autophagy and apoptosis in HUVECs.

MT2A plays an important role in maintaining intracellular zinc homeostasis because of its binding capacity with zinc, and zinc plays a key role in regulating apoptosis^17^. However, the proteins that interact with MT2A are largely unknown. Endothelial-overexpressed lipopolysaccharide (LPS)-associated factor 1 (EOLA1) interacts with MT2A in ECV304 cells^18^. This group also found that EOLA1 regulated MT2A to modulate HUVEC apoptosis during inflammatory responses^19^. In addition, in esophageal cancer cells, the interaction of esophageal cancer related gene 2 (ECRG2) and MT2A was suggested to play an important role in the carcinogenesis of esophageal cancer; ECRG2 co-localizing with MT2A was mostly in nuclei and slightly in cytoplasm^20^. In this study, we identified HMBOX1 as a new interaction protein of MT2A, and the interaction had an important role in control of intracellular free zinc level. Therefore, we provide a new point in the network of maintaining intracellular zinc homeostasis in HUVECs.

In normal cells, the intracellular free zinc level is maintained within a narrow range: zinc deficiency could induce cell apoptosis and overexpression could inhibit cell apoptosis^21^,^22^. Although the relationship between zinc and autophagy is not well known, zinc can positively regulate autophagy. Zinc is critical for basal and induced autophagy in MCF-7 breast cancer cells, astrocytes and human hepatoma cells^23^,^24^,^25^. Especially in PC12 cells, zinc-induced autophagy could facilitate cell survival^26^. We found that knockdown or overexpression of HMBOX1 decreased or elevated the intracellular free zinc level, respectively. Decreasing the level of zinc by TPEN reversed the apoptosis inhibition induced by HMBOX1 overexpression. Thus, HMBOX1 maintained basal autophagy in HUVECs to retain HUVEC survival by regulating intracellular zinc level. Our data suggest that HMBOX1 failed to regulate the intracellular free zinc level without MT2A. Only part of MT2A co-localized with HMBOX1 in HUVECs (Fig. 5B). MT2A proteins that do not interact with other proteins (free MT2A) in cytoplasm would bind zinc, because binding metals confers stability to MTs^27^. Therefore, in normal cells, some MT2A interacted with HMBOX1 and some free MT2A combined with zinc to maintain the intracellular free zinc at a moderate level. With HMBOX1 knockdown, the interacting MT2A proteins were separated to bind zinc in the cytoplasm, which decreased the intracellular free zinc level. HMBOX1 overexpression increased the direct interaction between HMBOX1 and MT2A; the free MT2A proteins interacted with HMBOX1 and released free zinc, for increased intracellular free zinc level and inhibited cell apoptosis.

Recent studies showed that zinc could inhibit the apoptosis of sheep pulmonary artery endothelial cells induced by LPS by decreasing the activity of caspase-3. Moreover, zinc inhibited the enzymatic activity of purified recombinant caspase-3 *in vitro*^28^,^29^. We found that the up-regulated free zinc level could suppress the level of cleaved caspase-3, which induced HUVEC apoptosis. In addition, overexpressing HMBOX1 inhibited the phosphorylation of 4EBP1 and p70S6K, downstream targets of mTOR, which could be abrogated by a zinc chelator, TPEN. Thus, autophagy promoted by HMBOX1 could depend on zinc and mTOR.

**Figure 8:**
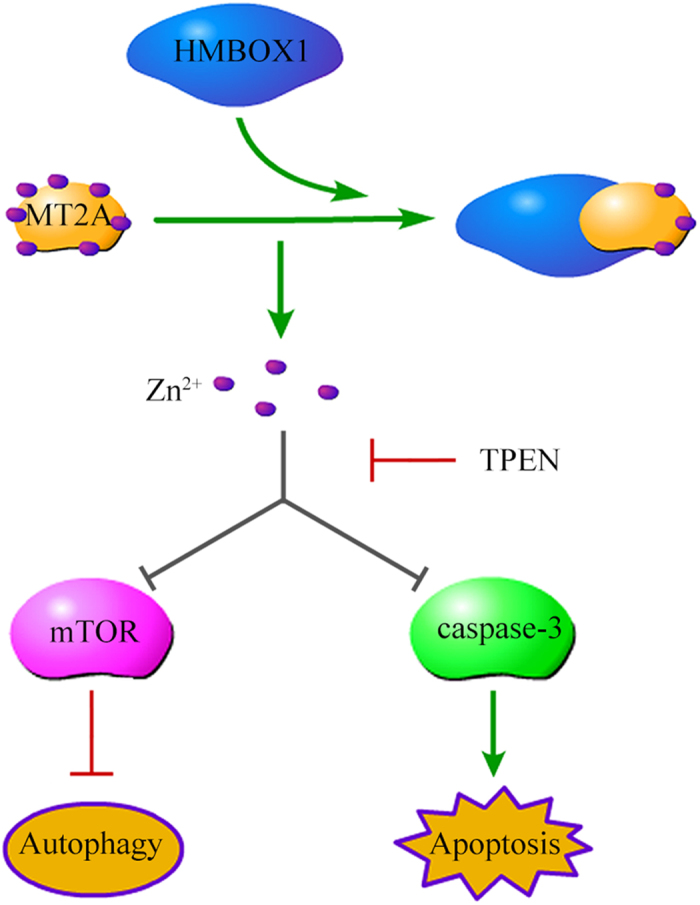
Working model of HMBOX1 effect in HUVECs. HMBOX1 interacted with MT2A to increase the intracellular level of free zinc in HUVECs, which promoted HUVEC autophagy by inactivating mTOR and inhibited HUVEC apoptosis by reducing the activity of caspase-3. TPEN reversed the pro-autophagy and anti-apoptotic effects of HMBOX1.

In summary, we show that a new factor, HMBOX1, is abundantly expressed in the cytoplasm of HUVECs. HMBOX1 maintained HUVEC survival by regulating both autophagy and apoptosis. Knocking down HMBOX1 level in HUVECs inhibited autophagy, and cells underwent apoptosis. HMBOX1 increased the intracellular free zinc level by interacting with MT2A. The up-regulated zinc level inhibited mTOR signaling and promoted HUVEC autophagy. Moreover, the altered zinc level affected the level of cleaved caspase-3 and thus inhibited HUVEC apoptosis induced by FGF-2 deprivation (Fig. 8). By identifying HMBOX1 as a regulator of intracellular free zinc, we demonstrate the mechanisms underlying HMBOX1 regulating apoptosis and autophagy in HUVECs.

## Methods

### Ethics statement

All experimental procedures and animal care were performed in accordance with the ARRIVE guidelines^30^ and approved by the ethics committee in Shandong University.

### Antibodies

Antibodies for HMBOX1 (sc-87768), GAPDH (sc-47724), GFP (sc-9996), caspase-3 (sc-7148), and horseradish peroxidase-conjugated secondary antibodies were all from Santa Cruz Biotechnology (Santa Cruz, CA). β-actin (A5441) and MT2A (SAB1402848) from Sigma-Aldrich (USA); antibodies for caspase-8 (9746), caspase-9 (9502), poly (ADP-ribose) polymerase (PARP, 9542S), LC3B (2775S), phospho-p70S6K (p-p70S6K; 9206S), p70S6K (9202S), p-4EBP1 (9459S), and 4EBP1 (9452S) were from Cell Signaling Technology (USA). Secondary antibodies for immunofluorescence were goat anti-rabbit IgG Alexa Fluor-488 (A-11008), donkey anti-mouse IgG Alexa Fluor-546 (A-10036) and goat anti-rat IgG Alexa Fluor-633 (A-21094, all Invitrogen).

### Cell culture and treatment

HUVECs were obtained as described^31^ and cultured on gelatin-coated plastic dishes in M199 medium (Gibco, 31100-035) supplemented with 10% fetal bovine serum (Hyclone, SV30087.02) and 2 ng/mL FGF-2 (EssexBio Group Zhuhai, China) in a humidified incubator at 37 °C with 5% CO_2_. HUVECs were used at passages 5 to 10. Confluent HUVECs were cultured in basal M199 medium without FGF-2 for the indicated time. HEK293 cells were obtained from the Cell Bank of the Chinese Academy of Sciences (Shanghai, China) and were grown in DMEM (Gibco, 12800–017) with 8% fetal bovine serum (FBS) (Hyclone, SV30087.02), penicillin (50 U/ml) and streptomycin (50 μg/ml) (Invitrogen, 10378–016). TPEN (P4413) and Bafilomycin A1 (B1793) purchased from Sigma-Aldrich (USA).

### Immunofluorescence assay

Immunofluorescence assay was performed as described^32^. Cells were fixed with 4% paraformaldehyde and blocked with normal goat serum (1:30) at room temperature, incubated with primary antibodies (1:100) overnight at 4 °C, then washed with phosphate buffered saline (PBS) and incubated with secondary antibodies (1:200) for 1 h at 37 °C in the dark. Nuclei were counterstained with DAPI. Zeiss LSM700 (Carl Zeiss Canada Ltd) for fluorescence detection at the indicated excitation wavelength. Carl Zeiss ZEN 2010 was used to measure fluorescence intensity in at least 10 regions for each labeling condition, with representative results shown.

### Cell apoptosis assay

Cells were stained with 5 μg/ml acridine orange (AO, Fluka) for 5 min at room temperature. Nuclear condensation and fragmentation were observed by laser scanning confocal microscopy (Leica, DMIRE2, Wetzlar, Germany). Cells were fixed in 4% formaldehyde for 10 min, then incubated with Hoechst 33258 (2 μg/ml) (Sigma, 94403) for 60 min at 37 °C. Stained cells were washed twice with PBS, then viewed under an Olympus (Japan) BH-2 fluorescence microscope. Cells were scored as apoptotic if nuclei were much brighter or showed condensed chromatin and nuclear fragmentation. In addition, apoptosis was measured by terminal deoxynucleotidyl transferase-mediated dUTP nick-end labeling (TUNEL) to detect *in situ* nuclear DNA fragmentation^33^. DNA fragmentation of treated cells was detected by use of the DeadEnd Fluorometric TUNEL System (Promega, TB235). Apoptosis was evaluated by laser scanning confocal microscopy (Leica, DMIRE2, Wetzlar, Germany). Cell apoptosis was also quantified using an AnnexinV-FITC/PI apoptosis kit (BioLegend, 640914) according to the manufacturer’s instructions. After treatment, cells were collected and rinsed with cell staining buffer (BioLegend, 420201) and then resuspended in 100 ul Annexin-binding buffer. The suspension was incubated with 8 μl Annexin V-FITC for 40min and then 2.5 μl propidium iodide (PI) for 10 min in the dark at RT. FITC or PI positive cells were determined using a flow cytometer (ImageStreamX MarkII, Amnis, USA). The fluorescence of FITC and PI were measured in the FL2 channel (488 nm) and FL4 channel (488 nm), respectively. The results were analyzed using IDEAS Application v6.0 software (Amnis, USA).

### Western blot analysis

Cell lysates were prepared in RIPA lysis buffer (Beyotime, P0013B) containing 1 mM PMSF. Western blot analysis was performed as described^34^. Equal amounts of proteins were run on 12% or 15% SDS-polyacrylamide gel. Proteins in gels were transferred to polyvinylidene difluoride membranes (Millipore, IPVH00010), which were blocked with 5% (w/v) nonfat dry milk in PBS-Tween 20 (PBST; 0.05%) for 1 h, then incubated with primary antibodies at 4 °C Covernight. Membranes were washed in PBST for 3 times and incubated with the combination of the horseradish peroxidase-conjugated secondary antibodies for 1 h at room temperature. Immunoreactive protein bands were developed by use of an enhanced chemiluminescence kit (Thermo Fisher, 34080). GAPDH or β-actin was used as loading control. The relative level of proteins was analyzed by use of ImageJ 1.47 (US National Institutes of Health).

### Yeast two-hybrid screen

Briefly, to express the bait protein, full-length human HMBOX1 cDNA was cloned into the yeast expression vector pGBKT7 (Clontech, 630489). The yeast strain AH109 (Clontech, 630444) was sequentially transformed with this bait plasmid and then a human liver cDNA library on a pACT2 vector (Clontech, 638822). In total, 4.8 × 10^5^ transformants were selectively screened by use of HIS3 (in the presence of 2.5 mM of 3-amino-1,2,4-triazole) as the primary reporter gene, then reporter genes ADE2 and lacZ to verify the positive colonies. The prey vectors in triple-positive yeast colonies were recovered and cDNA inserts were sequenced. For further validation of the interaction between HMBOX1 and the candidate proteins, pGBKT7-HMBOX1 and the library plasmids encoding the interacting partners were retransformed into AH109, and the activation of reporter genes was verified.

### Immunoprecipitation

Immunoprecipitation asaay was performed as described^35^. Briefly, HEK293 cells were washed with ice-cold PBS twice and lysed in western and immunoprecipitation buffer (Beyotime, P0013) containing 150 mM NaCl, 20 mM Tris–HCl (pH 7.5), 1% TritonX-100, and proteinase inhibitor PMSF mix. After centrifugation (12000 rpm) at 4 °C for 15 min, the supernatant was collected and precleared with protein A/G agarose beads (Beyotime, P2012) at 4 °C for 1 h. After centrifugation, supernatant was incubated with specific antibodies or normal IgG (as control), then Protein A/G beads overnight at 4 °C. The beads were washed 3 times with western and immunoprecipitation buffer, then eluted with 2 × SDS loading buffer. The immunoprecipitated proteins were examined by western blot assay.

### Determination of intracellular zinc concentration

Briefly, treated HUVECs were washed twice with PBS and incubated with 2.5 μM FluoZin-3 (Invitrogen, F-24195) in basic M199 medium at 37 °C for 30 min in the dark, then washed twice with PBS. The fluorescence of FluoZin-3 was evaluated under an Olympus (Japan) BH-2 fluorescence microscope, and data were analyzed by use of ImageJ 1.47. For flow cytometry, treated cells were trypsinized, centrifuged and resuspended in 2.5 μM FluoZin-3 in basic M199 medium (37 °C, 30 min) in the dark. Samples were analyzed by use of a FACS Calibur flow cytometer (BD Bioscience, FACS101). At least 10000 cells were collected. Data are from 3 independent experimental results and were analyzed by use of Flowjo 7.6.1 (Stanford University, USA). To confirm the zinc specificity of FluoZin-3, cells were simultaneously incubated with 25 μM TPEN, a zinc-specific chelator, for 30 min.

### RNA interference (RNAi) and overexpression

To knock down the expression of HMBOX1 or MT2A, RNA interference was performed as described^7^. siRNA against HMBOX1 was designed and synthesized by Invitrogen (USA), and siRNA against MT2A (sc-93491) was from Santa Cruz Biotechnology (Santa Cruz, CA). HUVECs at 60% to 70% confluence were transfected with HMBOX1 or MT2A siRNA (20–80 nM) for 24 h by use of RNAiFect Transfection Reagent according to the manufacturer’s instructions (QIAGEN, 301605), then gene silencing was assessed by western blot or immunofluorescence assay. Scramble siRNA was used as a control (Santa Cruz Biotechnology, sc-37007). The cDNA constructs encoding wild-type HMBOX1 subunit (pCMV-HMBOX1) (Origene, SC319800), empty vector (pRC-CMV), pEGFP or pEGFP-MT2A (Origene, RG202748) were transfected by use of Lipofectamine 2000 reagent (Invitrogen, 11668–019).

### Statistical analysis

All experiments were repeated at least 3 times independently. Data are expressed as mean ± SEM and were analyzed by one-way ANOVA with use of SPSS v11.5 (SPSS Inc., Chicago, IL). Images were processed by use of Graphpad Prism 5 (GraphPad Software, La Jolla, CA, USA) and Adobe Photoshop CC (Adobe, San Jose, USA). *P* < 0.05 was considered statistically significant.

## Additional Information

**How to cite this article**: Ma, H.L. *et al.* HMBOX1 interacts with MT2A to regulate autophagy and apoptosis in vascular endothelial cells. *Sci. Rep.* **5**, 15121; doi: 10.1038/srep15121 (2015).

## Supplementary Material

Supplementary Information
