# Supplementary material for: HMBOX1 interacts with MT2A to regulate autophagy and apoptosis in vascular endothelial cells
Source: Sci Rep. 2015 Oct 12;5:15121. doi: 10.1038/srep15121 (PMC4600982; doi:10.1038/srep15121)
Supplement: Supplementary Information [file srep15121-s1.pdf]

HMBOX1 interacts with MT2A to regulate autophagy and apoptosis in vascular endothelial cells

HanLin Ma[1], Le Su[1], HongWei Yue[1], XiaoLei Yin[1], Jing Zhao[1], ShangLi Zhang[1], HsiangFu Kung[1, 3], ZhiGang Xu[1,*], JunYing Miao[1, 2, *]

[1] *Shandong Provincial Key Laboratory of Animal Cells and Developmental Biology, School of Life Science, Shandong University, Jinan 250100, China*

[2] *The Key Laboratory of Cardiovascular Remodeling and Function Research, Chinese Ministry of Education and Chinese Ministry of Health, Shandong University Qilu Hospital, Jinan, 250012, China*

[3] *Institute of Pathology and Southwest Cancer Center, Third Military Medical University, Chongqing, 400038, China.*

* Correspondence to: JunYing Miao and ZhiGang Xu, Shandong Provincial Key Laboratory of Animal Cells and Developmental Biology, School of Life Science, Shandong University, Jinan 250100, China.

Fax: + 86 531 88565610; Tel.: + 86 531 88364929.

E-mail address: miaojy@sdu.edu.cn, xuzg@sdu.edu.cn

HanLin Ma and Le Su have contributed equally to this work

Supplementary Information

Supplementary Figures

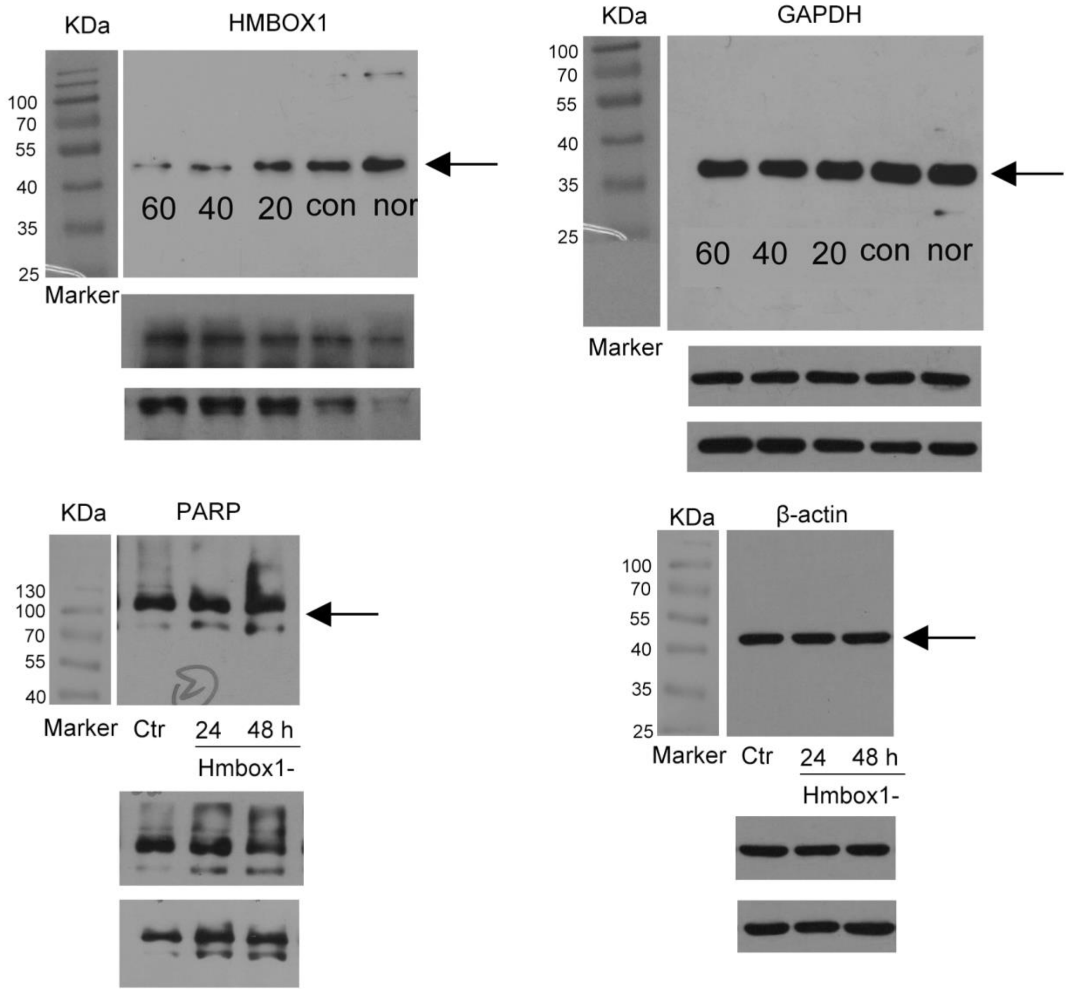

Figure S1. Uncropped blots probed with HMBOX1, GAPDH, PARP, and β-actin.

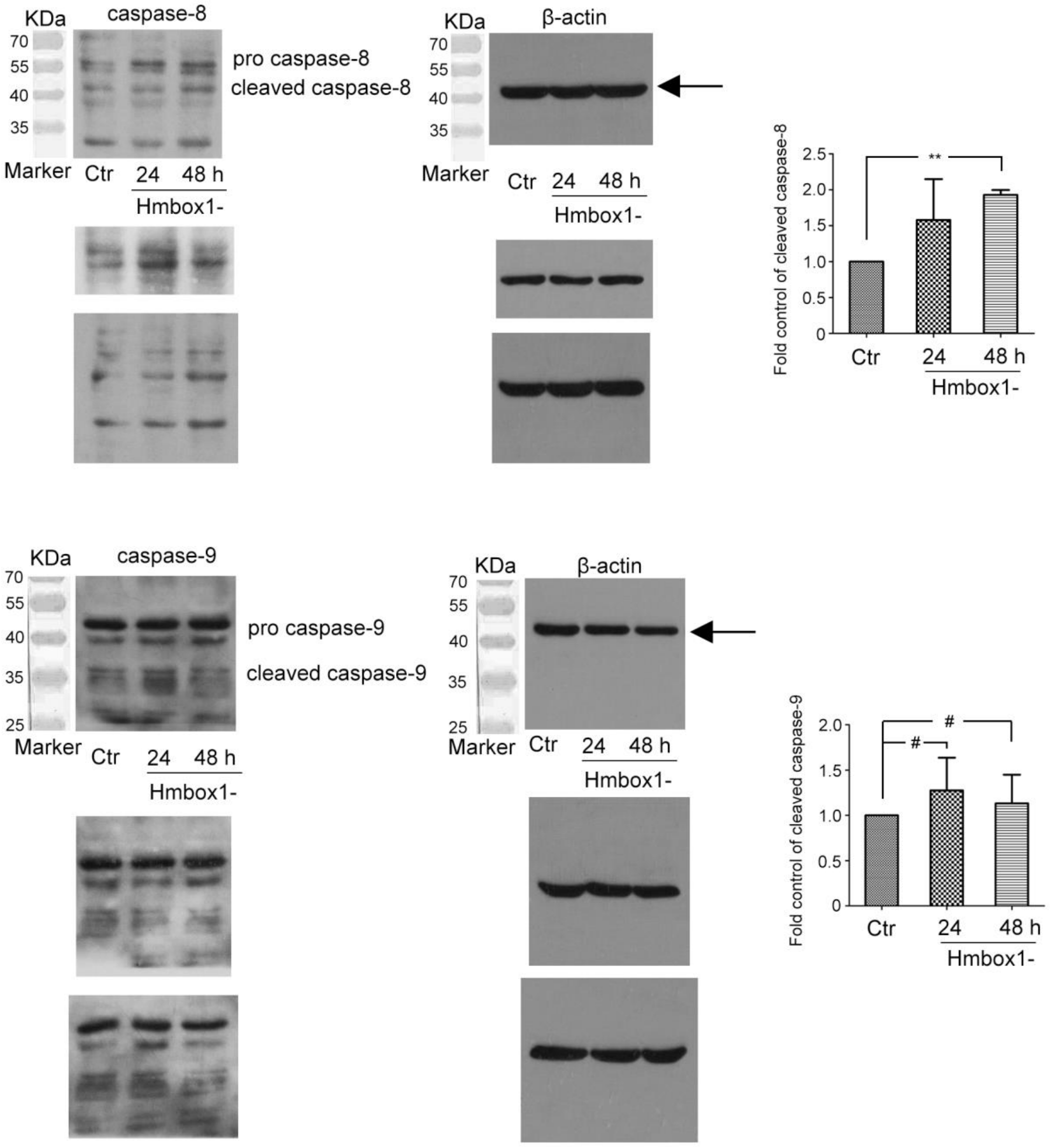

Figure S2. Uncropped blots probed with caspase-8,caspase-9 and β-actin. Western blot analysis of cleaved forms of caspase-8 and caspase-9 in HUVECs transfected with HMBOX1 siRNA for 24 or 48 h. The level of cleaved caspase-8 and caspase-9 was relative to that of β-actin. (Data are mean ± SEM, #$p > 0.05$, **$P < 0.01$, n = 3).

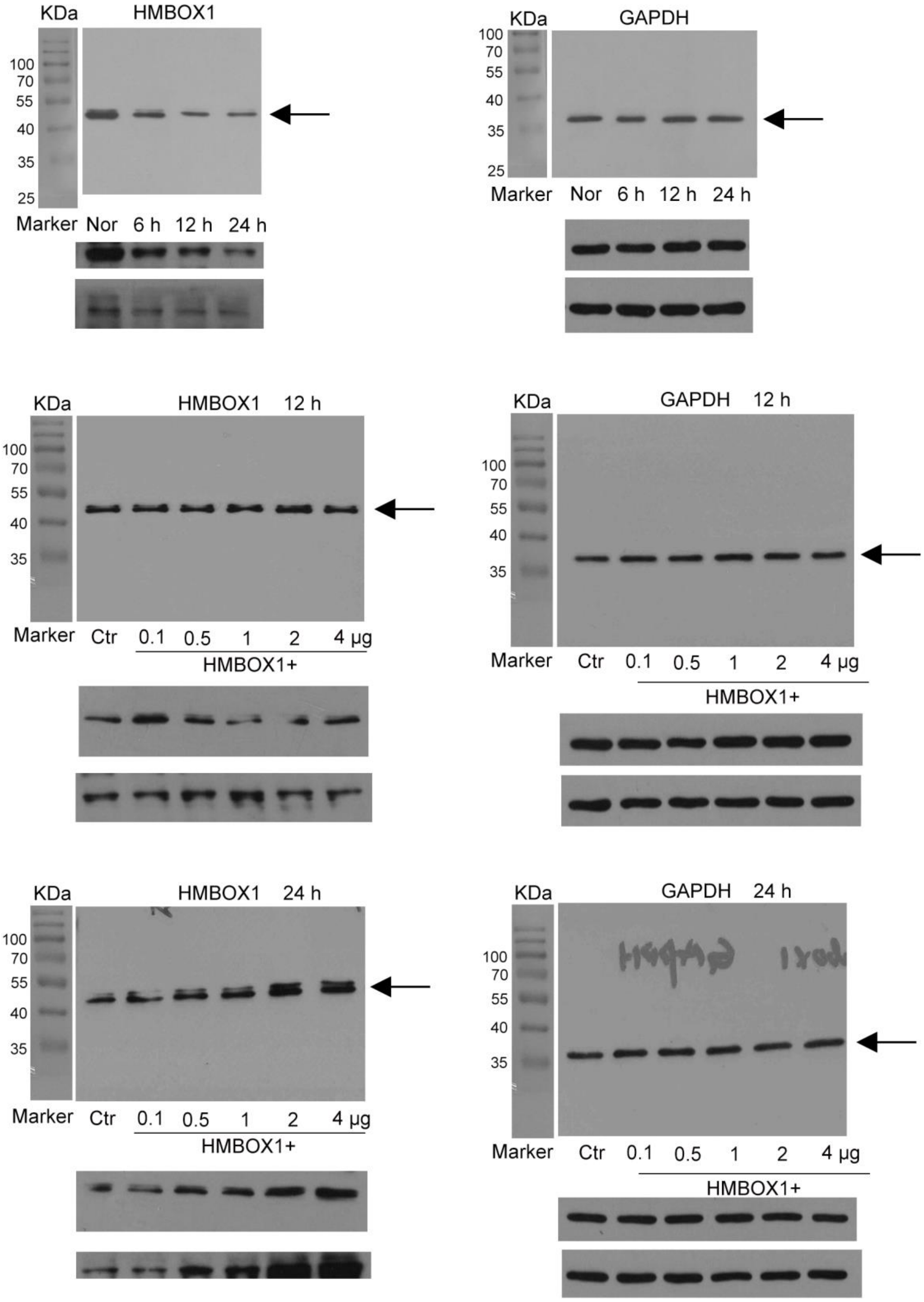

Figure S3. Uncropped blots probed with HMBOX1 and GAPDH.

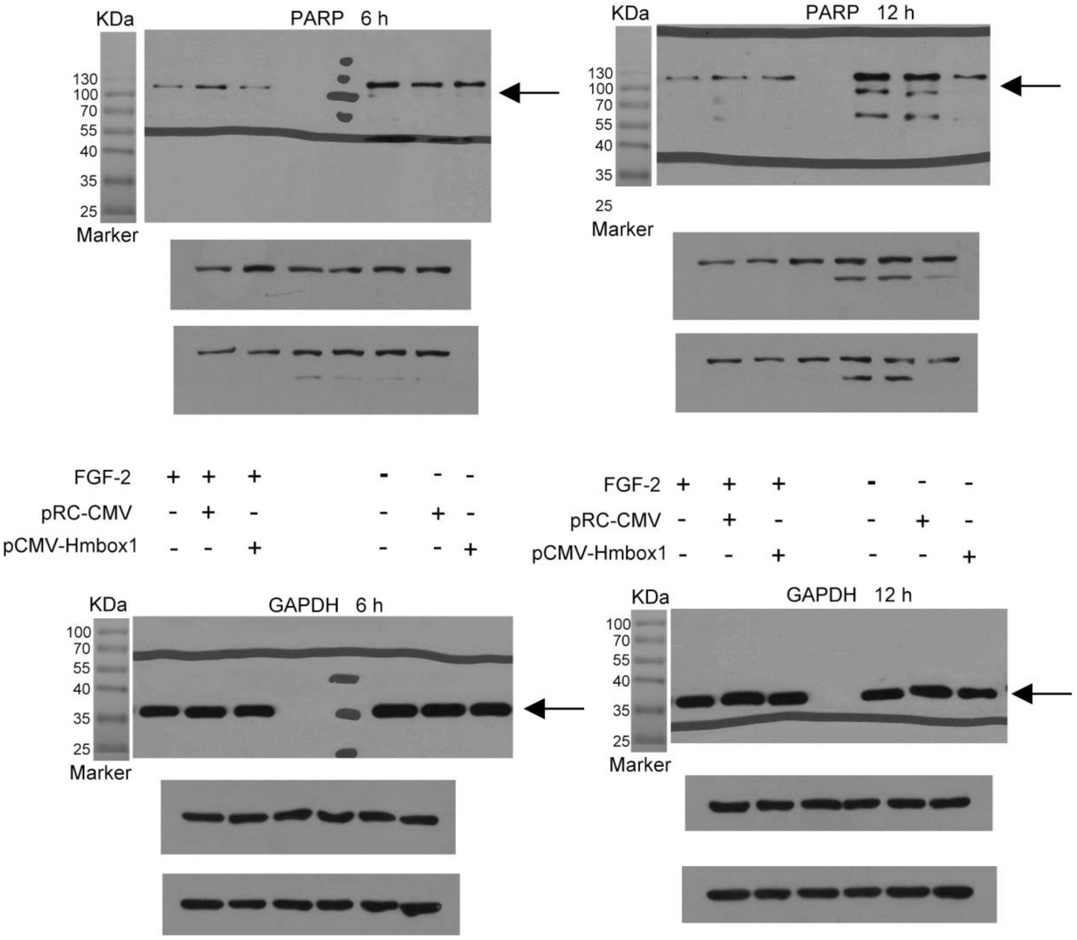

Figure S4. Uncropped blots probed with GAPDH and PARP.

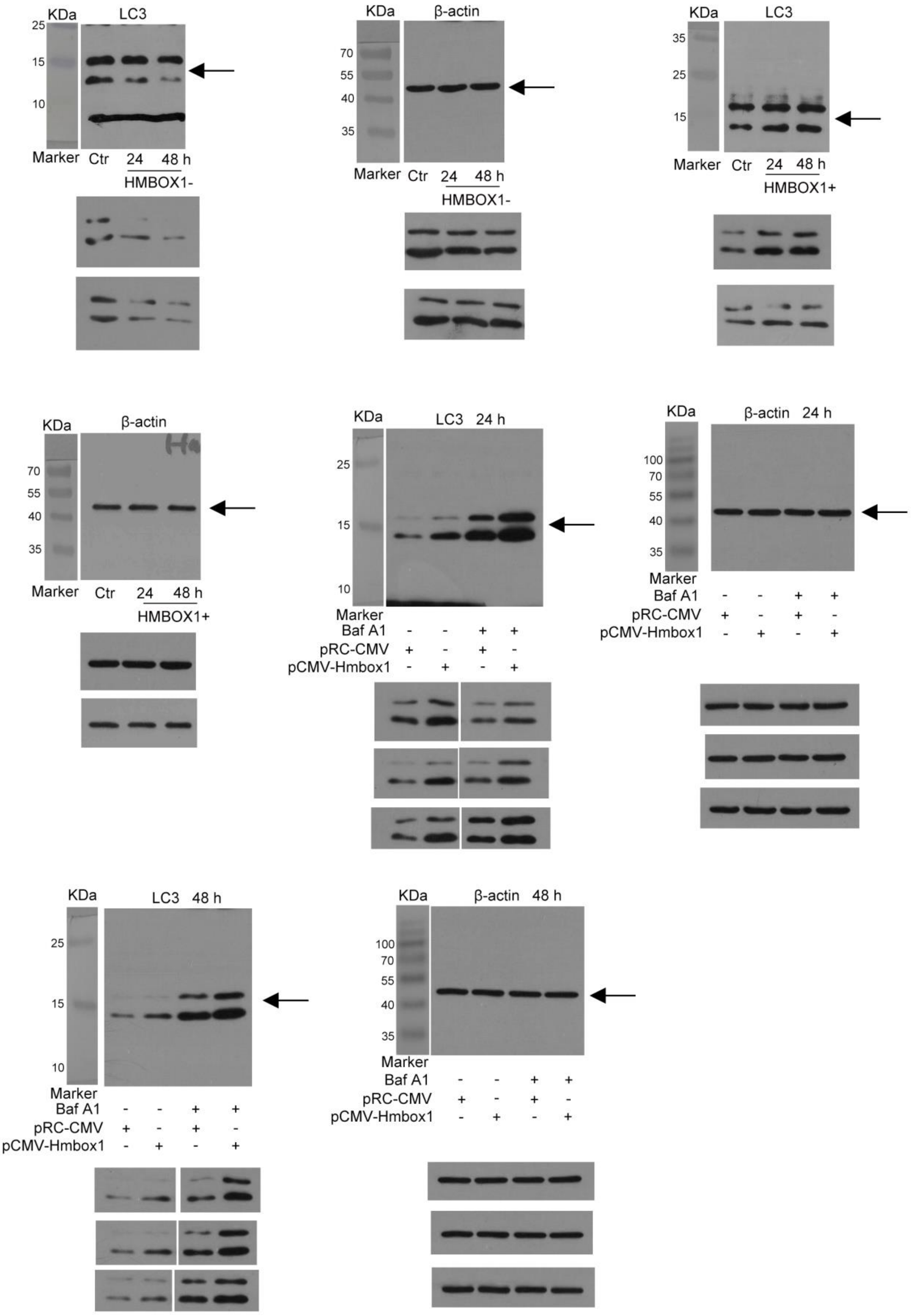

Figure S5. Uncropped blots probed with LC3 and β-actin.

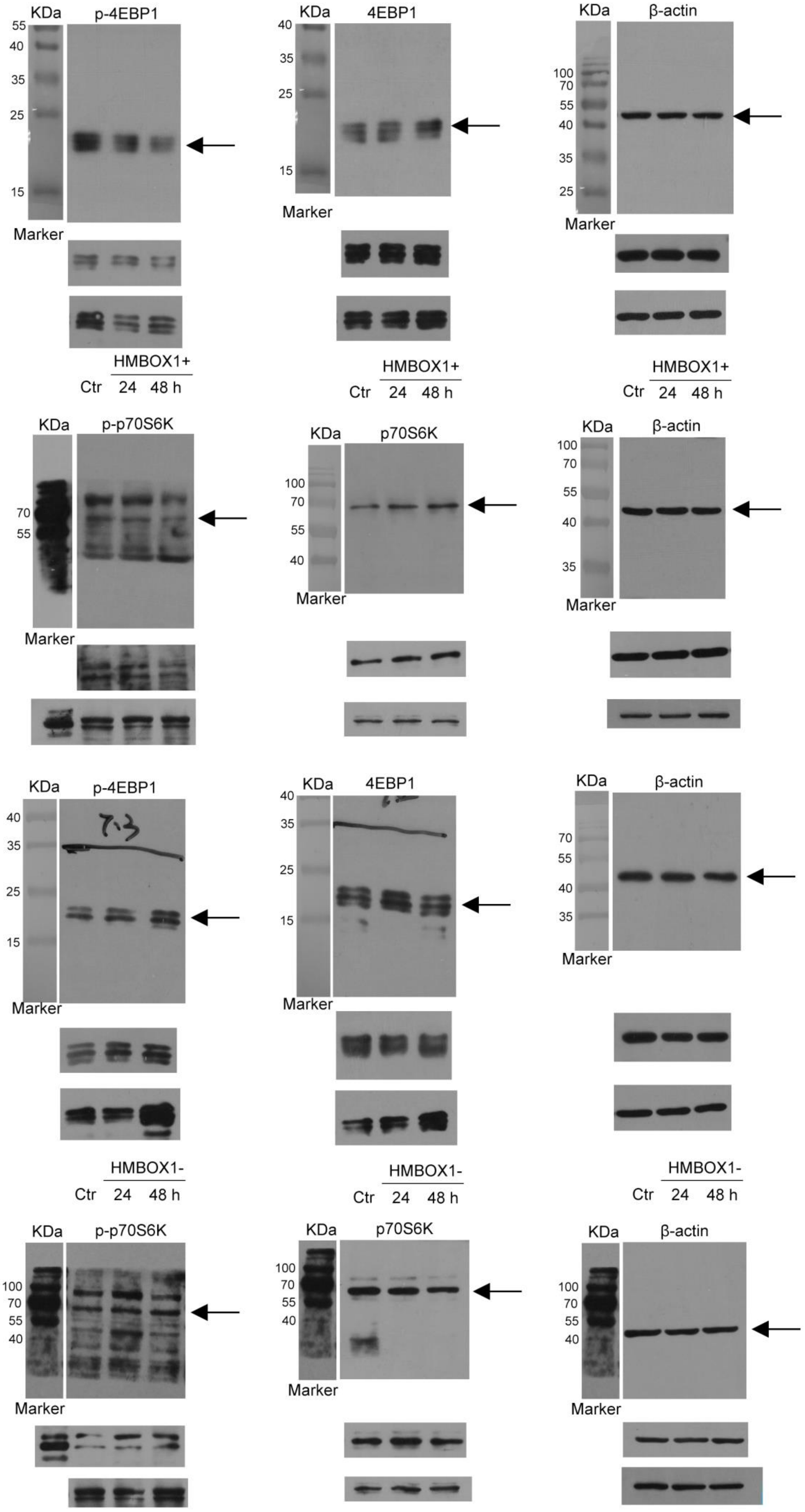
KDa
p-4EBP1
4EBP1
β-actin
Marker
HMBOX1+
Ctr
24
48 h
p-p70S6K
p70S6K
HMBOX1-
7-3
100
70
55
40
35
25
15

Figure S6. Uncropped blots probed with p-4EBP1, 4EBP1, p-p70S6K, p70S6K and β-actin.

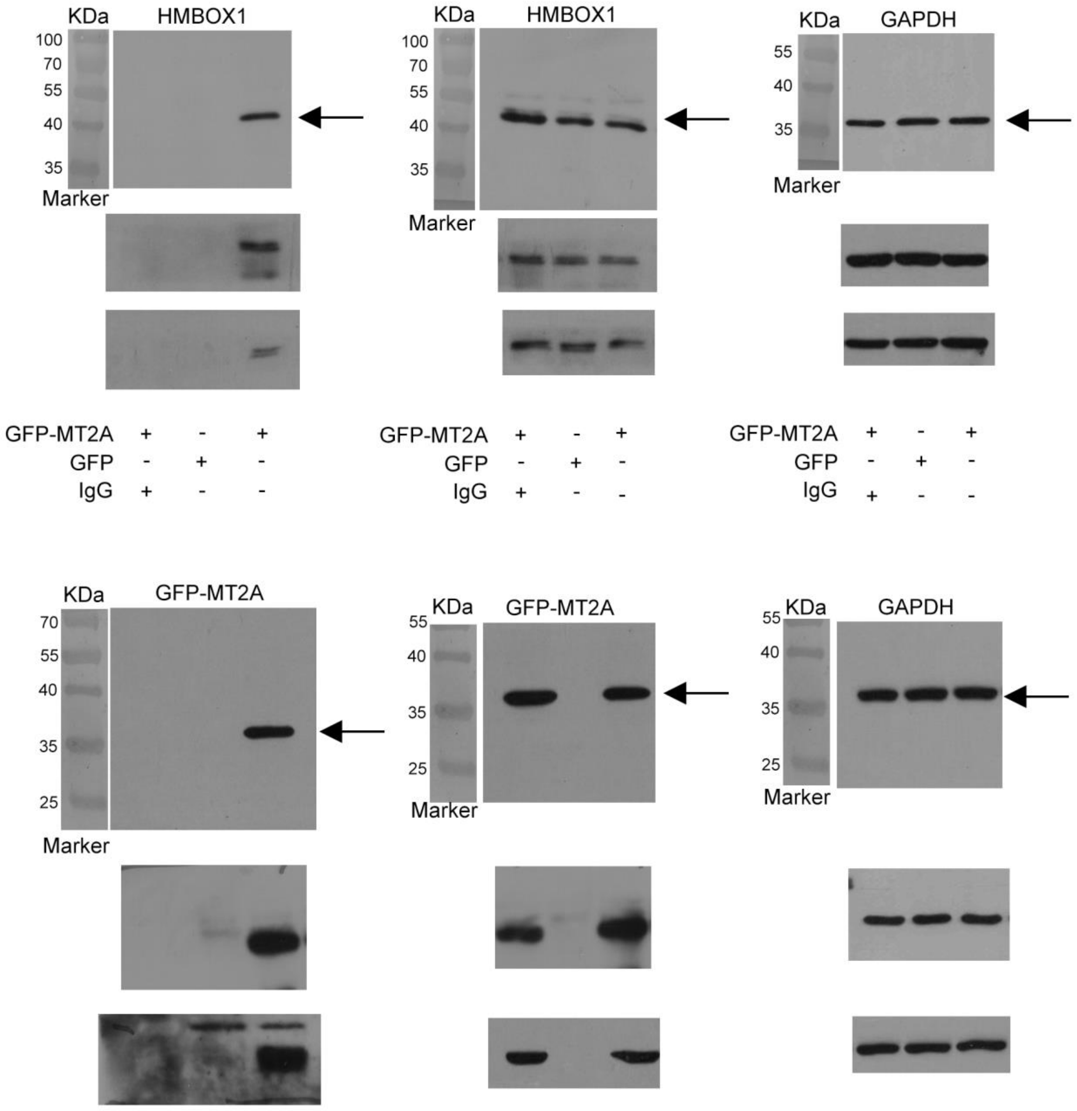

Figure S7. Uncropped blots probed with HMBOX1, MT2A and GAPDH.

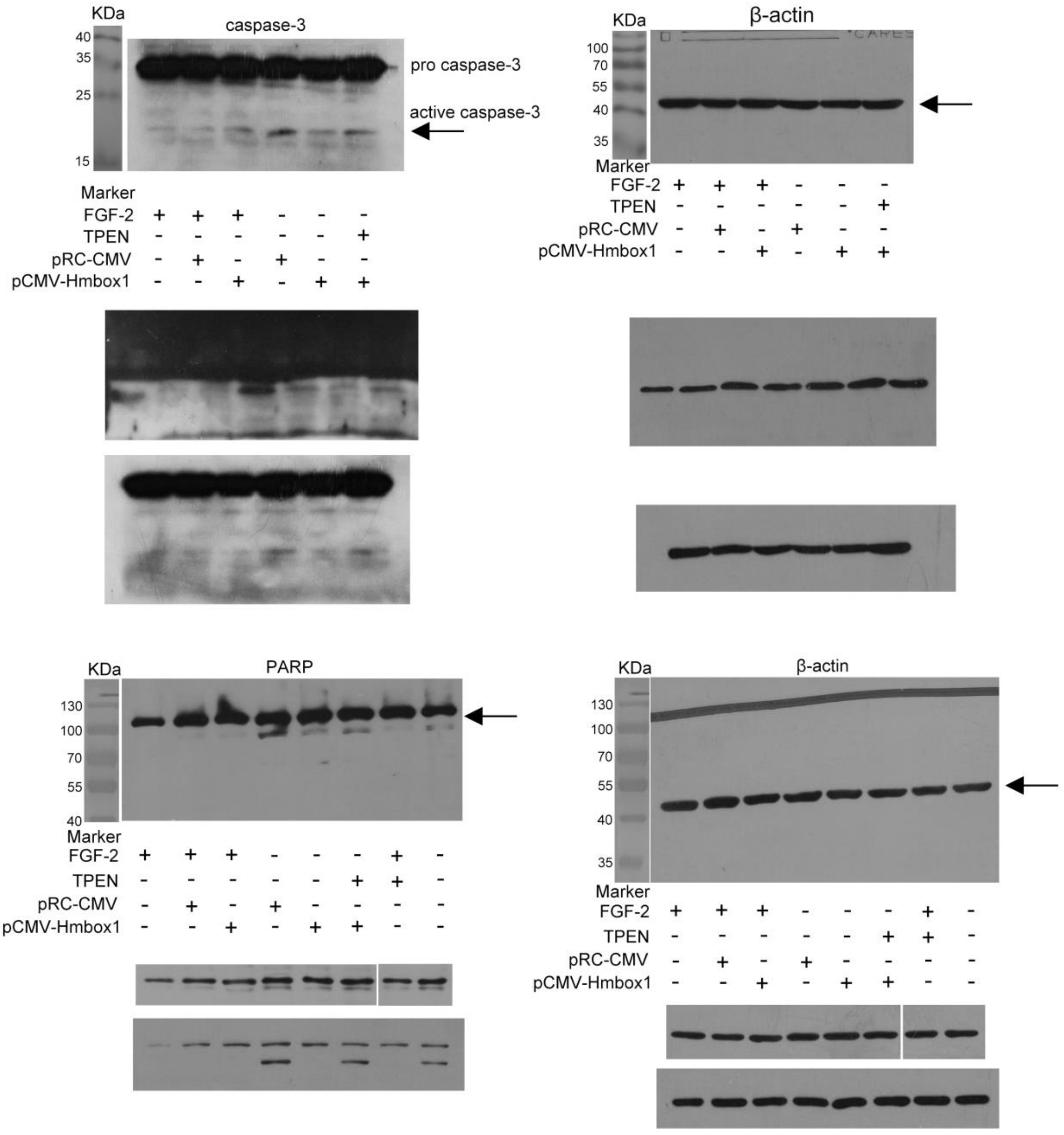

Figure S8. Uncropped blots probed with Caspase-3, PARP and β-actin.

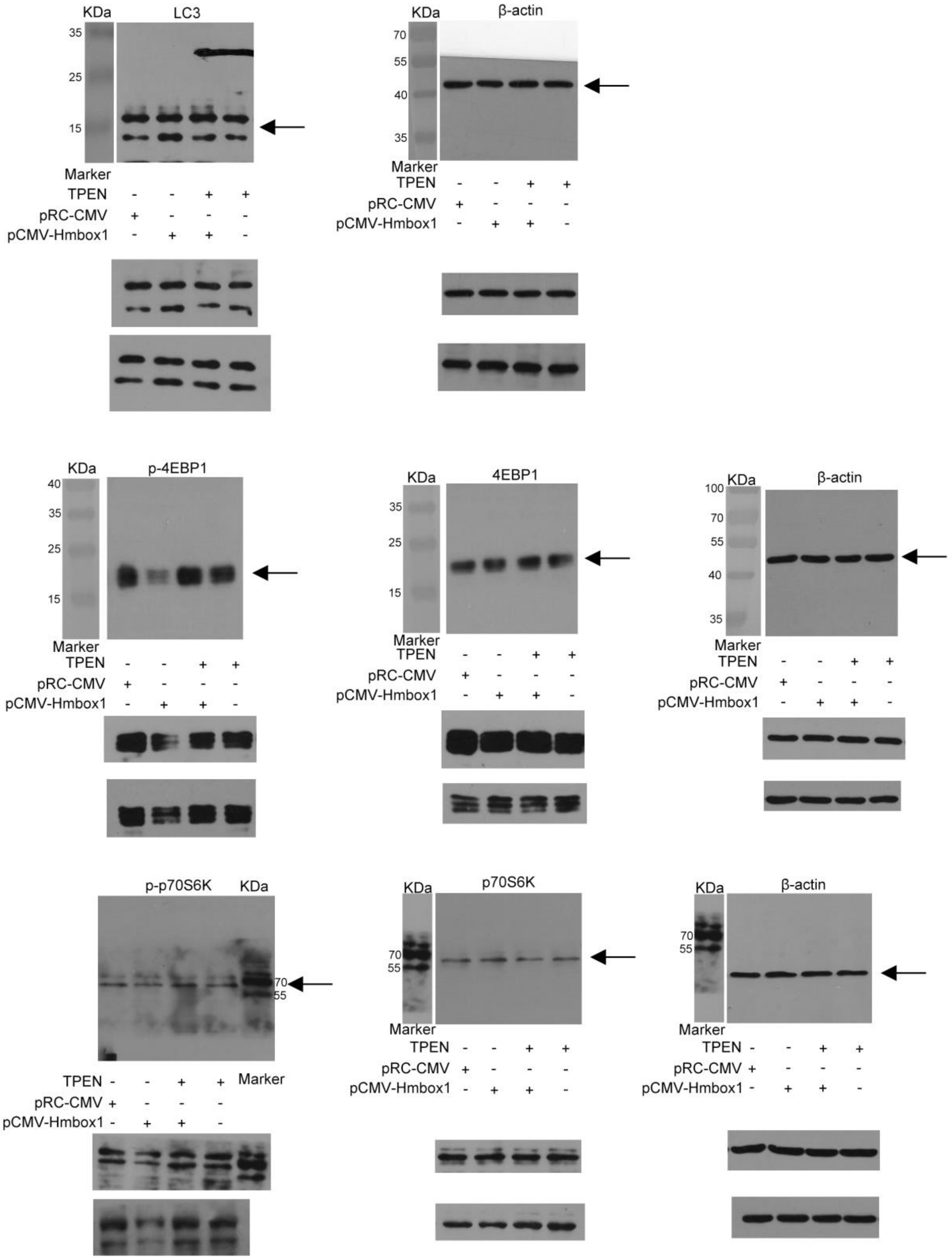

Figure S9. Uncropped blots probed with LC3, β-actin, p-4EBP1, 4EBP1, p-p70S6K and p70S6K.

Supplementary Tables

Yeast transformation	Transformation efficiency[a] (CFU/μg library)	Total transformants[b]	$His^+/Ade^+/Laz^{+c}$	True positive
	2.4×10^3	5×10^5	30 colonies	pACT2-MT2A (10 colonies)

Table S1. Yeast two-hybrid screening with HMBOX1 as bait

HMBOX1 gene was cloned into the GAL4 DNA binding domain construct NpGBKT7 as bait for screening a human liver cDNA library. Transformation efficiency was 2.4×10^3, yielding up to 5×10^5 independent transformants; 140 clones grew in the YPD medium deficient in tryptophan, leucine, histidine, and adenine; 30 showed β-galactosidase activity. Sequencing of these pACT2 prey plasmids identified MT2A as the HMBOX1-interacting protein with 10 repeated clones. a: Transformation efficiency = Total transformants/amount of library DNA in micrograms. b: Total transformants = (colonies/plate)/(volume/plate)×(volume of total reaction/dilution factor). c: β-galactosidase activity of the positive colonies.
